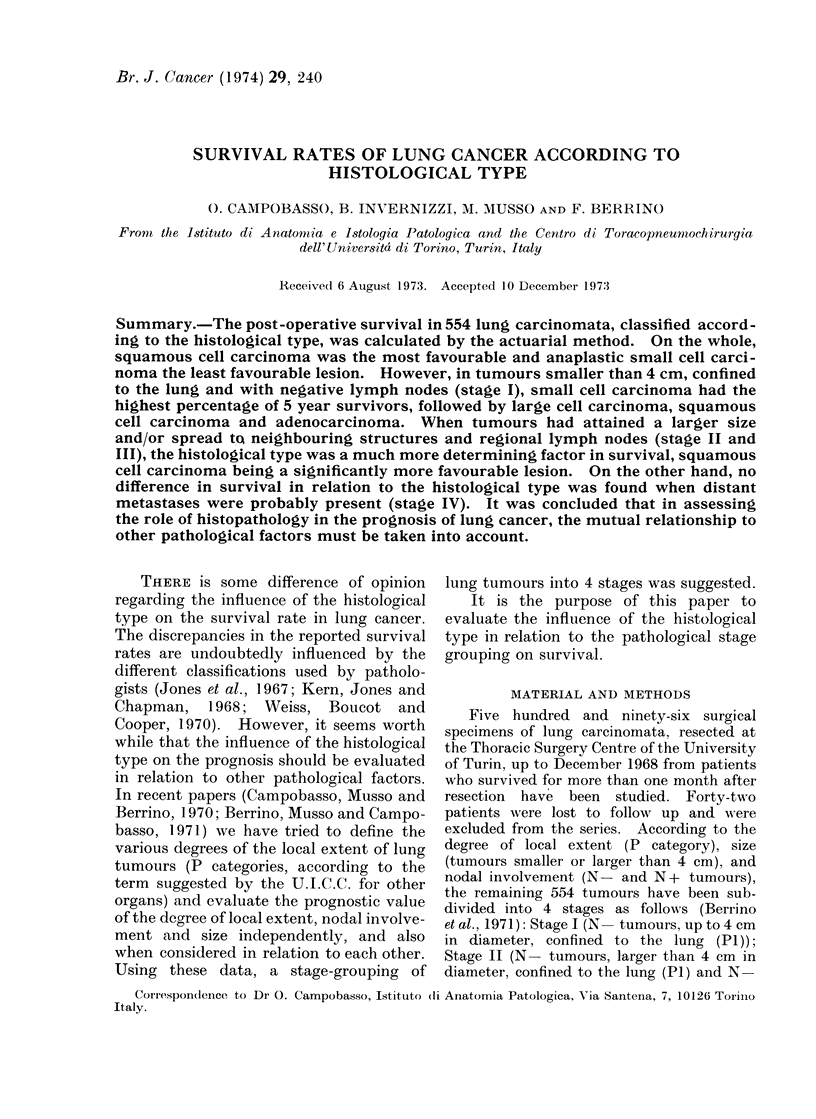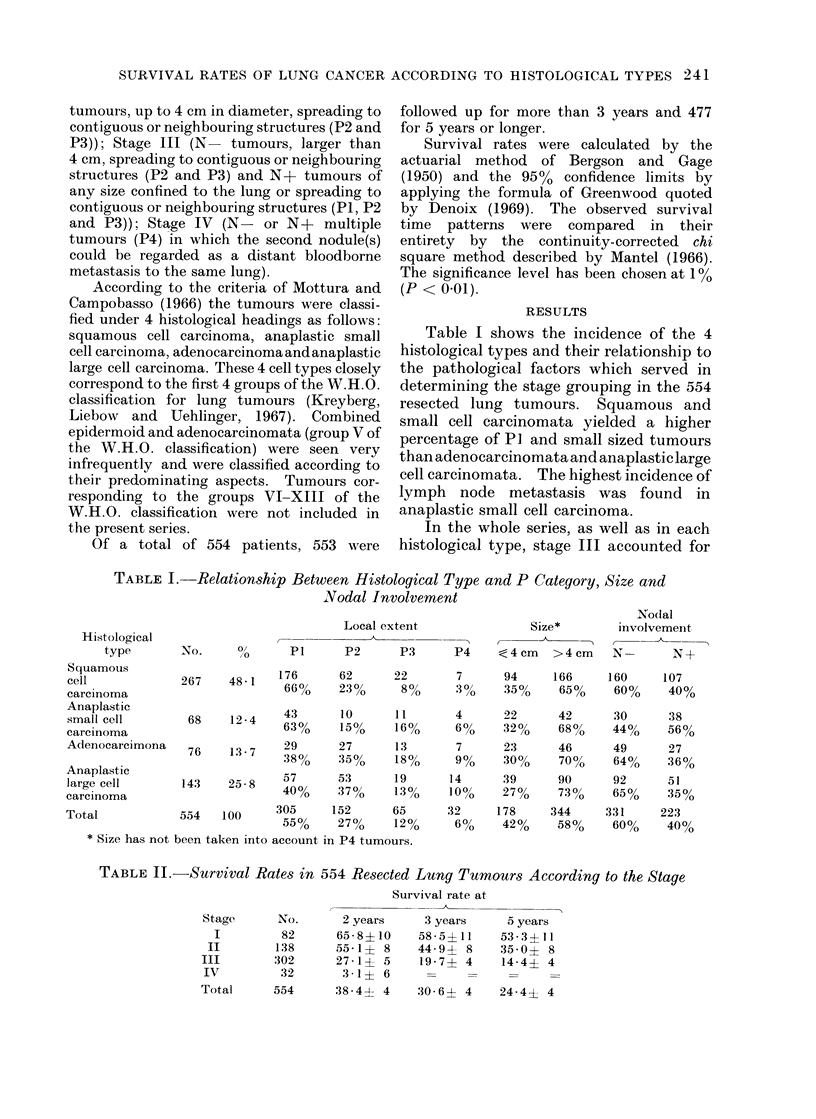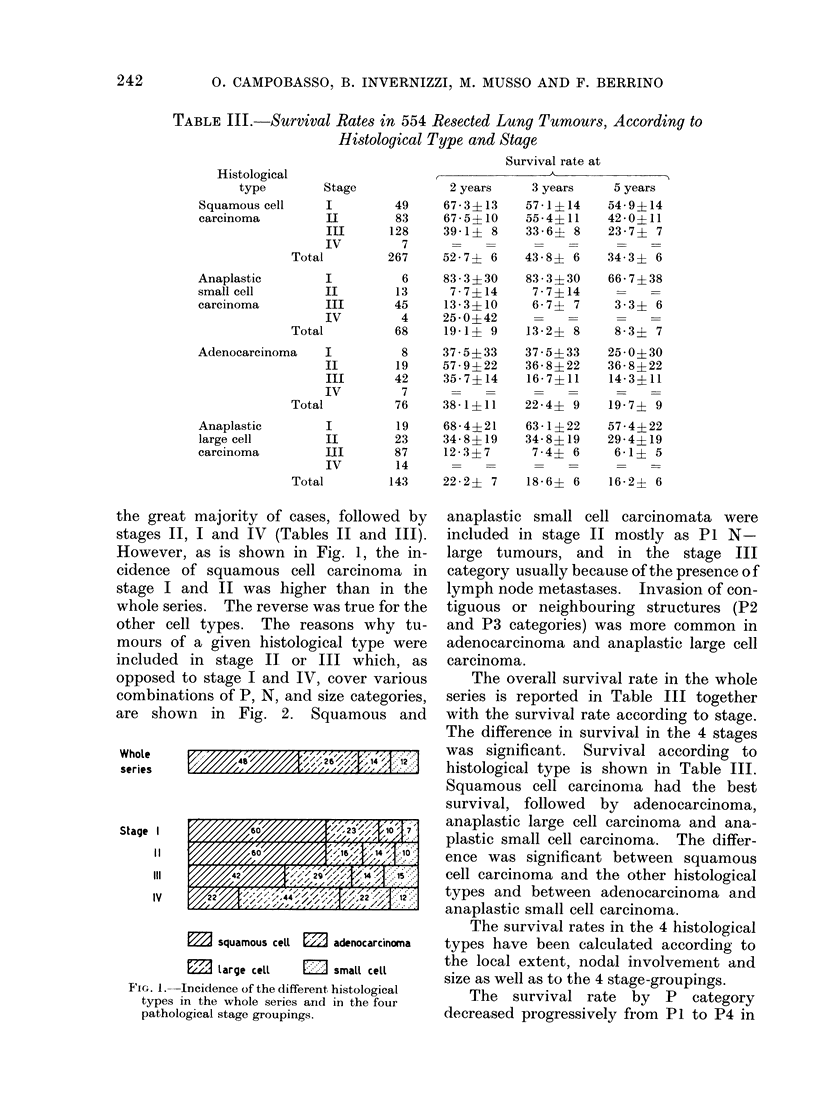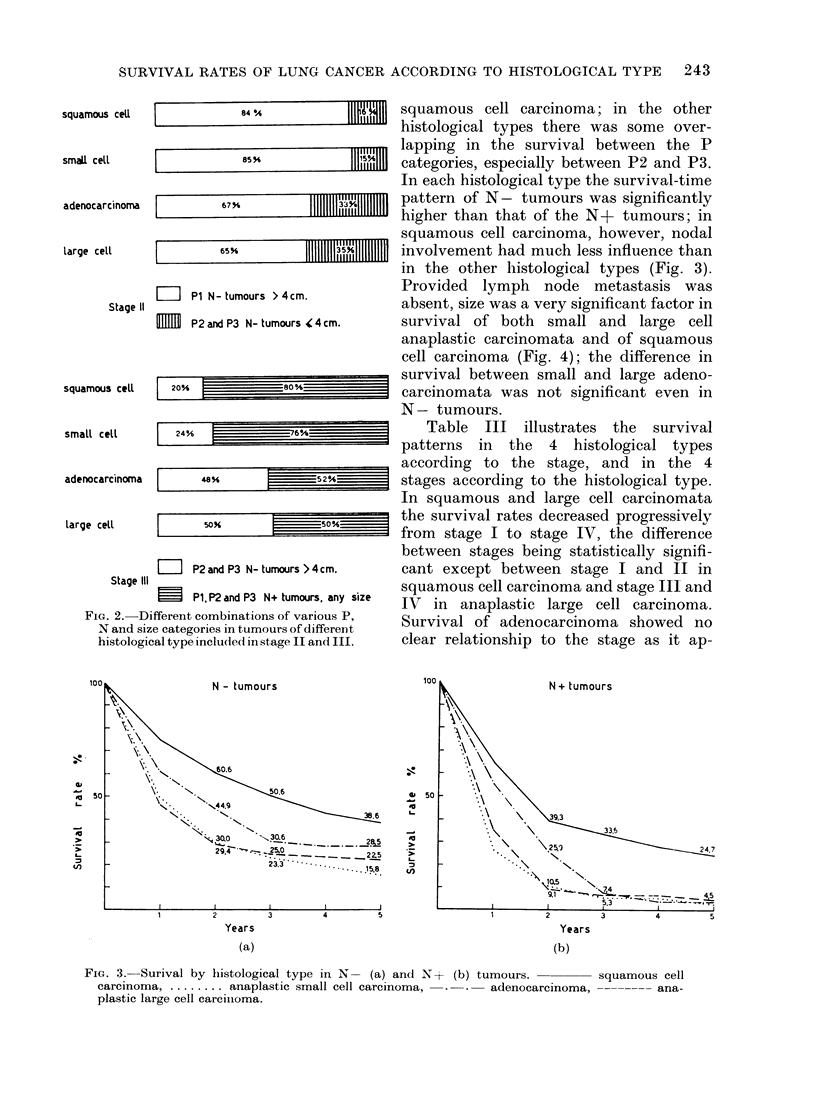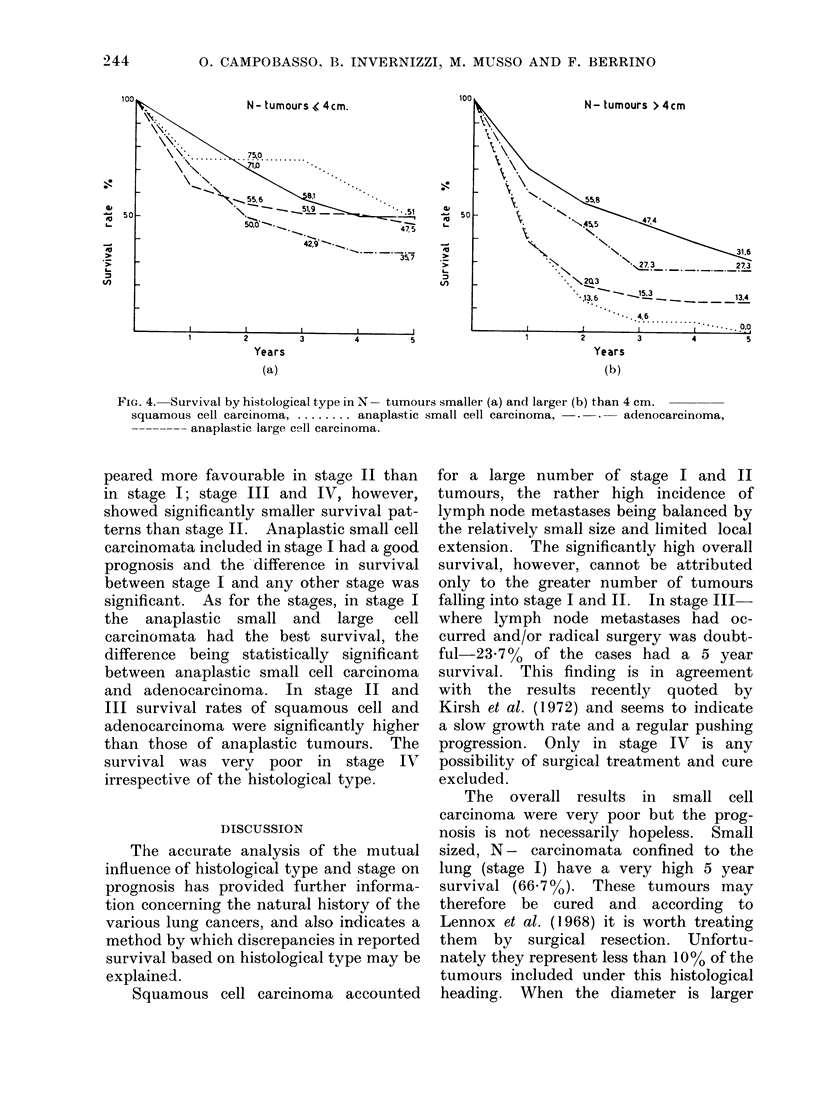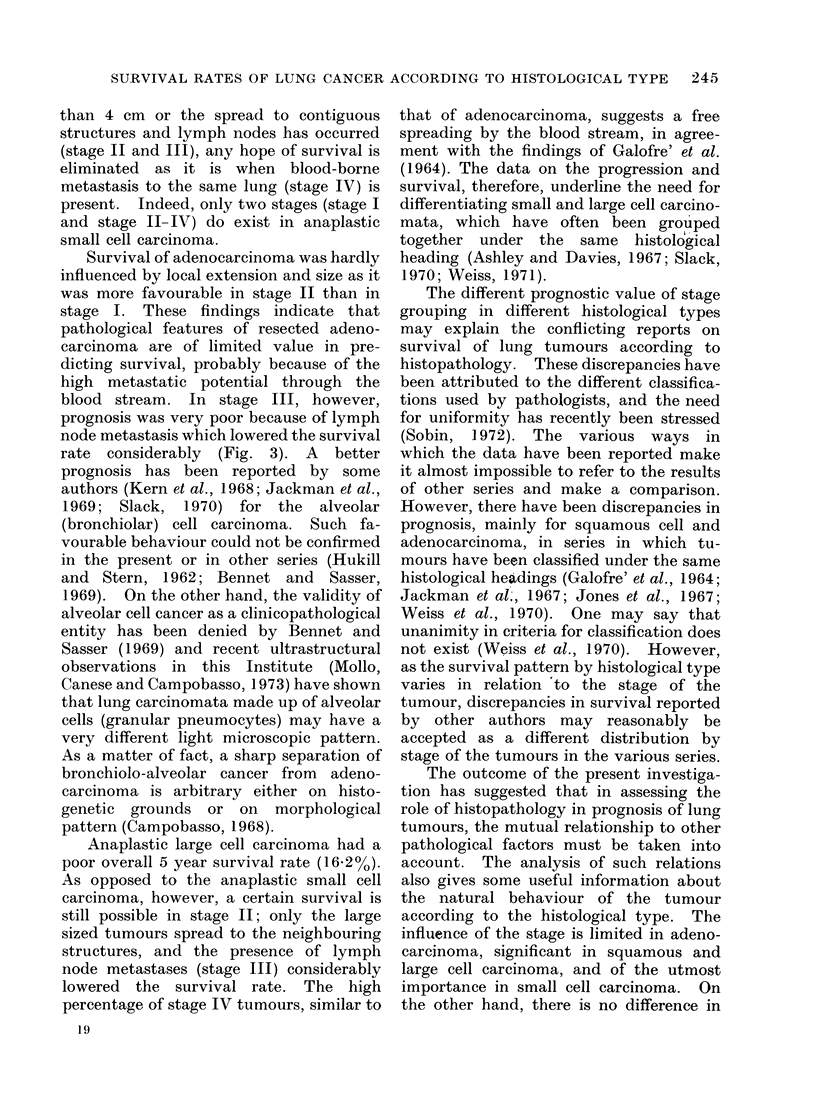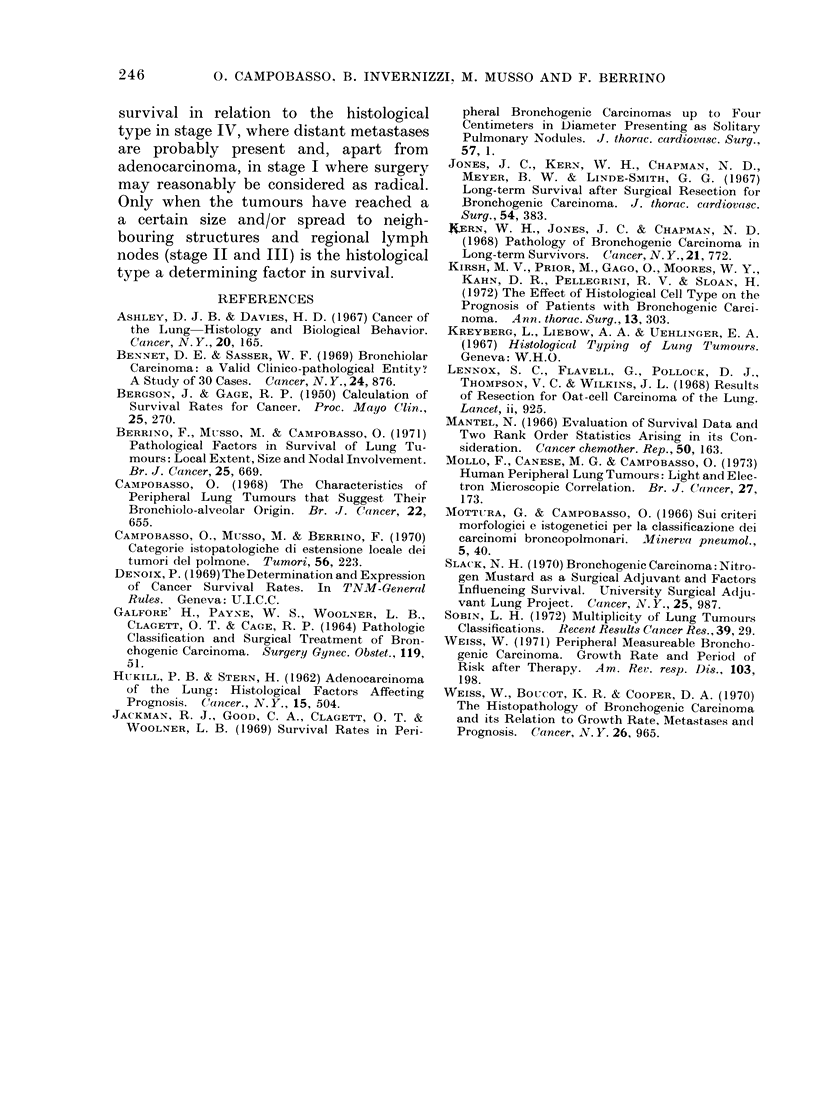# Survival Rates of Lung Cancer According to Histological Type

**DOI:** 10.1038/bjc.1974.63

**Published:** 1974-03

**Authors:** O. Campobasso, B. Invernizzi, M. Musso, F. Berrino

## Abstract

The post-operative survival in 554 lung carcinomata, classified according to the histological type, was calculated by the actuarial method. On the whole, squamous cell carcinoma was the most favourable and anaplastic small cell carcinoma the least favourable lesion. However, in tumours smaller than 4 cm, confined to the lung and with negative lymph nodes (stage I), small cell carcinoma had the highest percentage of 5 year survivors, followed by large cell carcinoma, squamous cell carcinoma and adenocarcinoma. When tumours had attained a larger size and/or spread to neighbouring structures and regional lymph nodes (stage II and III), the histological type was a much more determining factor in survival, squamous cell carcinoma being a significantly more favourable lesion. On the other hand, no difference in survival in relation to the histological type was found when distant metastases were probably present (stage IV). It was concluded that in assessing the role of histopathology in the prognosis of lung cancer, the mutual relationship to other pathological factors must be taken into account.


Br. J. Cancer (1974) 29, 240

### SURVIVAL RATES OF LUNG CANCER ACCORDING TO HISTOLOGICAL TYPE

O. CAMPOBASSO, B. INVERNIZZI, M. MUSSO AND F. BERRINO

*From the Istituto di Anatomia e Istologia Patologica and the Centro di Toracopneumochirurgia dell'Università di Torino, Turin, Italy*

Received 6 August 1973. Accepted 10 December 1973

**Summary.—The post-operative survival in 554 lung carcinomata, classified according to the histological type, was calculated by the actuarial method. On the whole, squamous cell carcinoma was the most favourable and anaplastic small cell carcinoma the least favourable lesion. However, in tumours smaller than 4 cm, confined to the lung and with negative lymph nodes (stage I), small cell carcinoma had the highest percentage of 5 year survivors, followed by large cell carcinoma, squamous cell carcinoma and adenocarcinoma. When tumours had attained a larger size and/or spread to neighbouring structures and regional lymph nodes (stage II and III), the histological type was a much more determining factor in survival, squamous cell carcinoma being a significantly more favourable lesion. On the other hand, no difference in survival in relation to the histological type was found when distant metastases were probably present (stage IV). It was concluded that in assessing the role of histopathology in the prognosis of lung cancer, the mutual relationship to other pathological factors must be taken into account.**

THERE is some difference of opinion regarding the influence of the histological type on the survival rate in lung cancer. The discrepancies in the reported survival rates are undoubtedly influenced by the different classifications used by pathologists (Jones *et al.*, 1967; Kern, Jones and Chapman, 1968; Weiss, Boucot and Cooper, 1970). However, it seems worth while that the influence of the histological type on the prognosis should be evaluated in relation to other pathological factors. In recent papers (Campobasso, Musso and Berrino, 1970; Berrino, Musso and Campobasso, 1971) we have tried to define the various degrees of the local extent of lung tumours (P categories, according to the term suggested by the U.I.C.C. for other organs) and evaluate the prognostic value of the degree of local extent, nodal involvement and size independently, and also when considered in relation to each other. Using these data, a stage-grouping of lung tumours into 4 stages was suggested.

It is the purpose of this paper to evaluate the influence of the histological type in relation to the pathological stage grouping on survival.

### MATERIAL AND METHODS

Five hundred and ninety-six surgical specimens of lung carcinomata, resected at the Thoracic Surgery Centre of the University of Turin, up to December 1968 from patients who survived for more than one month after resection have been studied. Forty-two patients were lost to follow up and were excluded from the series. According to the degree of local extent (P category), size (tumours smaller or larger than 4 cm), and nodal involvement (N— and N+ tumours), the remaining 554 tumours have been subdivided into 4 stages as follows (Berrino *et al.*, 1971): Stage I (N— tumours, up to 4 cm in diameter, confined to the lung (P1)); Stage II (N— tumours, larger than 4 cm in diameter, confined to the lung (P1) and N—

Correspondence to Dr O. Campobasso, Istituto di Anatomia Patologica, Via Santena, 7, 10126 Torino Italy.

tumours, up to 4 cm in diameter, spreading to contiguous or neighbouring structures (P2 and P3)); Stage III (N− tumours, larger than 4 cm, spreading to contiguous or neighbouring structures (P2 and P3) and N+ tumours of any size confined to the lung or spreading to contiguous or neighbouring structures (P1, P2 and P3)); Stage IV (N− or N+ multiple tumours (P4) in which the second nodule(s) could be regarded as a distant bloodborne metastasis to the same lung).

According to the criteria of Mottura and Campobasso (1966) the tumours were classified under 4 histological headings as follows: squamous cell carcinoma, anaplastic small cell carcinoma, adenocarcinoma and anaplastic large cell carcinoma. These 4 cell types closely correspond to the first 4 groups of the W.H.O. classification for lung tumours (Kreyberg, Liebow and Uehlinger, 1967). Combined epidermoid and adenocarcinomata (group V of the W.H.O. classification) were seen very infrequently and were classified according to their predominating aspects. Tumours corresponding to the groups VI–XIII of the W.H.O. classification were not included in the present series.

Of a total of 554 patients, 553 were followed up for more than 3 years and 477 for 5 years or longer.

Survival rates were calculated by the actuarial method of Bergson and Gage (1950) and the 95% confidence limits by applying the formula of Greenwood quoted by Denoix (1969). The observed survival time patterns were compared in their entirety by the continuity-corrected *chi* square method described by Mantel (1966). The significance level has been chosen at 1% ($P < 0{\cdot}01$).

### RESULTS

Table I shows the incidence of the 4 histological types and their relationship to the pathological factors which served in determining the stage grouping in the 554 resected lung tumours. Squamous and small cell carcinomata yielded a higher percentage of P1 and small sized tumours than adenocarcinomata and anaplastic large cell carcinomata. The highest incidence of lymph node metastasis was found in anaplastic small cell carcinoma.

In the whole series, as well as in each histological type, stage III accounted for

TABLE I.—*Relationship Between Histological Type and P Category, Size and Nodal Involvement*

| Histological type | No. | % | Local extent P1 | P2 | P3 | P4 | Size* ≤4 cm | >4 cm | Nodal involvement N− | N+ |
|---|---|---|---|---|---|---|---|---|---|---|
| Squamous cell carcinoma | 267 | 48·1 | 176<br>66% | 62<br>23% | 22<br>8% | 7<br>3% | 94<br>35% | 166<br>65% | 160<br>60% | 107<br>40% |
| Anaplastic small cell carcinoma | 68 | 12·4 | 43<br>63% | 10<br>15% | 11<br>16% | 4<br>6% | 22<br>32% | 42<br>68% | 30<br>44% | 38<br>56% |
| Adenocarcimona | 76 | 13·7 | 29<br>38% | 27<br>35% | 13<br>18% | 7<br>9% | 23<br>30% | 46<br>70% | 49<br>64% | 27<br>36% |
| Anaplastic large cell carcinoma | 143 | 25·8 | 57<br>40% | 53<br>37% | 19<br>13% | 14<br>10% | 39<br>27% | 90<br>73% | 92<br>65% | 51<br>35% |
| Total | 554 | 100 | 305<br>55% | 152<br>27% | 65<br>12% | 32<br>6% | 178<br>42% | 344<br>58% | 331<br>60% | 223<br>40% |

* Size has not been taken into account in P4 tumours.

TABLE II.—*Survival Rates in 554 Resected Lung Tumours According to the Stage*

| Stage | No. | Survival rate at 2 years | 3 years | 5 years |
|---|---|---|---|---|
| I | 82 | 65·8±10 | 58·5±11 | 53·3±11 |
| II | 138 | 55·1± 8 | 44·9± 8 | 35·0± 8 |
| III | 302 | 27·1± 5 | 19·7± 4 | 14·4± 4 |
| IV | 32 | 3·1± 6 | = = | = = |
| Total | 554 | 38·4± 4 | 30·6± 4 | 24·4± 4 |

TABLE III.—*Survival Rates in 554 Resected Lung Tumours, According to Histological Type and Stage*

| Histological type | Stage | | Survival rate at 2 years | 3 years | 5 years |
|---|---|---|---|---|---|
| Squamous cell carcinoma | I | 49 | 67·3±13 | 57·1±14 | 54·9±14 |
| | II | 83 | 67·5±10 | 55·4±11 | 42·0±11 |
| | III | 128 | 39·1± 8 | 33·6± 8 | 23·7± 7 |
| | IV | 7 | = = | = = | = = |
| | Total | 267 | 52·7± 6 | 43·8± 6 | 34·3± 6 |
| Anaplastic small cell carcinoma | I | 6 | 83·3±30 | 83·3±30 | 66·7±38 |
| | II | 13 | 7·7±14 | 7·7±14 | = = |
| | III | 45 | 13·3±10 | 6·7± 7 | 3·3± 6 |
| | IV | 4 | 25·0±42 | = = | = = |
| | Total | 68 | 19·1± 9 | 13·2± 8 | 8·3± 7 |
| Adenocarcinoma | I | 8 | 37·5±33 | 37·5±33 | 25·0±30 |
| | II | 19 | 57·9±22 | 36·8±22 | 36·8±22 |
| | III | 42 | 35·7±14 | 16·7±11 | 14·3±11 |
| | IV | 7 | = = | = = | = = |
| | Total | 76 | 38·1±11 | 22·4± 9 | 19·7± 9 |
| Anaplastic large cell carcinoma | I | 19 | 68·4±21 | 63·1±22 | 57·4±22 |
| | II | 23 | 34·8±19 | 34·8±19 | 29·4±19 |
| | III | 87 | 12·3±7 | 7·4± 6 | 6·1± 5 |
| | IV | 14 | = = | = = | = = |
| | Total | 143 | 22·2± 7 | 18·6± 6 | 16·2± 6 |

the great majority of cases, followed by stages II, I and IV (Tables II and III). However, as is shown in Fig. 1, the incidence of squamous cell carcinoma in stage I and II was higher than in the whole series. The reverse was true for the other cell types. The reasons why tumours of a given histological type were included in stage II or III which, as opposed to stage I and IV, cover various combinations of P, N, and size categories, are shown in Fig. 2. Squamous and anaplastic small cell carcinomata were included in stage II mostly as P1 N− large tumours, and in the stage III category usually because of the presence of lymph node metastases. Invasion of contiguous or neighbouring structures (P2 and P3 categories) was more common in adenocarcinoma and anaplastic large cell carcinoma.

FIG. 1.—Incidence of the different histological types in the whole series and in the four pathological stage groupings.

The overall survival rate in the whole series is reported in Table III together with the survival rate according to stage. The difference in survival in the 4 stages was significant. Survival according to histological type is shown in Table III. Squamous cell carcinoma had the best survival, followed by adenocarcinoma, anaplastic large cell carcinoma and anaplastic small cell carcinoma. The difference was significant between squamous cell carcinoma and the other histological types and between adenocarcinoma and anaplastic small cell carcinoma.

The survival rates in the 4 histological types have been calculated according to the local extent, nodal involvement and size as well as to the 4 stage-groupings.

The survival rate by P category decreased progressively from P1 to P4 in

FIG. 2.—Different combinations of various P, N and size categories in tumours of different histological type included in stage II and III.

squamous cell carcinoma; in the other histological types there was some overlapping in the survival between the P categories, especially between P2 and P3. In each histological type the survival-time pattern of N− tumours was significantly higher than that of the N+ tumours; in squamous cell carcinoma, however, nodal involvement had much less influence than in the other histological types (Fig. 3). Provided lymph node metastasis was absent, size was a very significant factor in survival of both small and large cell anaplastic carcinomata and of squamous cell carcinoma (Fig. 4); the difference in survival between small and large adenocarcinomata was not significant even in N− tumours.

Table III illustrates the survival patterns in the 4 histological types according to the stage, and in the 4 stages according to the histological type. In squamous and large cell carcinomata the survival rates decreased progressively from stage I to stage IV, the difference between stages being statistically significant except between stage I and II in squamous cell carcinoma and stage III and IV in anaplastic large cell carcinoma. Survival of adenocarcinoma showed no clear relationship to the stage as it ap-

FIG. 3.—Surival by histological type in N− (a) and N+ (b) tumours. ———— squamous cell carcinoma, ........ anaplastic small cell carcinoma, —·—·— adenocarcinoma, -------- anaplastic large cell carcinoma.

FIG. 4.—Survival by histological type in N− tumours smaller (a) and larger (b) than 4 cm. ———— squamous cell carcinoma, ........ anaplastic small cell carcinoma, —·—·— adenocarcinoma, -------- anaplastic large cell carcinoma.

peared more favourable in stage II than in stage I; stage III and IV, however, showed significantly smaller survival patterns than stage II. Anaplastic small cell carcinomata included in stage I had a good prognosis and the difference in survival between stage I and any other stage was significant. As for the stages, in stage I the anaplastic small and large cell carcinomata had the best survival, the difference being statistically significant between anaplastic small cell carcinoma and adenocarcinoma. In stage II and III survival rates of squamous cell and adenocarcinoma were significantly higher than those of anaplastic tumours. The survival was very poor in stage IV irrespective of the histological type.

### DISCUSSION

The accurate analysis of the mutual influence of histological type and stage on prognosis has provided further information concerning the natural history of the various lung cancers, and also indicates a method by which discrepancies in reported survival based on histological type may be explained.

Squamous cell carcinoma accounted for a large number of stage I and II tumours, the rather high incidence of lymph node metastases being balanced by the relatively small size and limited local extension. The significantly high overall survival, however, cannot be attributed only to the greater number of tumours falling into stage I and II. In stage III—where lymph node metastases had occurred and/or radical surgery was doubtful—23·7% of the cases had a 5 year survival. This finding is in agreement with the results recently quoted by Kirsh *et al.* (1972) and seems to indicate a slow growth rate and a regular pushing progression. Only in stage IV is any possibility of surgical treatment and cure excluded.

The overall results in small cell carcinoma were very poor but the prognosis is not necessarily hopeless. Small sized, N− carcinomata confined to the lung (stage I) have a very high 5 year survival (66·7%). These tumours may therefore be cured and according to Lennox *et al.* (1968) it is worth treating them by surgical resection. Unfortunately they represent less than 10% of the tumours included under this histological heading. When the diameter is larger than 4 cm or the spread to contiguous structures and lymph nodes has occurred (stage II and III), any hope of survival is eliminated as it is when blood-borne metastasis to the same lung (stage IV) is present. Indeed, only two stages (stage I and stage II–IV) do exist in anaplastic small cell carcinoma.

Survival of adenocarcinoma was hardly influenced by local extension and size as it was more favourable in stage II than in stage I. These findings indicate that pathological features of resected adenocarcinoma are of limited value in predicting survival, probably because of the high metastatic potential through the blood stream. In stage III, however, prognosis was very poor because of lymph node metastasis which lowered the survival rate considerably (Fig. 3). A better prognosis has been reported by some authors (Kern *et al.*, 1968; Jackman *et al.*, 1969; Slack, 1970) for the alveolar (bronchiolar) cell carcinoma. Such favourable behaviour could not be confirmed in the present or in other series (Hukill and Stern, 1962; Bennet and Sasser, 1969). On the other hand, the validity of alveolar cell cancer as a clinicopathological entity has been denied by Bennet and Sasser (1969) and recent ultrastructural observations in this Institute (Mollo, Canese and Campobasso, 1973) have shown that lung carcinomata made up of alveolar cells (granular pneumocytes) may have a very different light microscopic pattern. As a matter of fact, a sharp separation of bronchiolo-alveolar cancer from adenocarcinoma is arbitrary either on histogenetic grounds or on morphological pattern (Campobasso, 1968).

Anaplastic large cell carcinoma had a poor overall 5 year survival rate (16·2%). As opposed to the anaplastic small cell carcinoma, however, a certain survival is still possible in stage II; only the large sized tumours spread to the neighbouring structures, and the presence of lymph node metastases (stage III) considerably lowered the survival rate. The high percentage of stage IV tumours, similar to that of adenocarcinoma, suggests a free spreading by the blood stream, in agreement with the findings of Galofre' *et al.* (1964). The data on the progression and survival, therefore, underline the need for differentiating small and large cell carcinomata, which have often been grouped together under the same histological heading (Ashley and Davies, 1967; Slack, 1970; Weiss, 1971).

The different prognostic value of stage grouping in different histological types may explain the conflicting reports on survival of lung tumours according to histopathology. These discrepancies have been attributed to the different classifications used by pathologists, and the need for uniformity has recently been stressed (Sobin, 1972). The various ways in which the data have been reported make it almost impossible to refer to the results of other series and make a comparison. However, there have been discrepancies in prognosis, mainly for squamous cell and adenocarcinoma, in series in which tumours have been classified under the same histological headings (Galofre' *et al.*, 1964; Jackman *et al.*, 1967; Jones *et al.*, 1967; Weiss *et al.*, 1970). One may say that unanimity in criteria for classification does not exist (Weiss *et al.*, 1970). However, as the survival pattern by histological type varies in relation to the stage of the tumour, discrepancies in survival reported by other authors may reasonably be accepted as a different distribution by stage of the tumours in the various series.

The outcome of the present investigation has suggested that in assessing the role of histopathology in prognosis of lung tumours, the mutual relationship to other pathological factors must be taken into account. The analysis of such relations also gives some useful information about the natural behaviour of the tumour according to the histological type. The influence of the stage is limited in adenocarcinoma, significant in squamous and large cell carcinoma, and of the utmost importance in small cell carcinoma. On the other hand, there is no difference in survival in relation to the histological type in stage IV, where distant metastases are probably present and, apart from adenocarcinoma, in stage I where surgery may reasonably be considered as radical. Only when the tumours have reached a a certain size and/or spread to neighbouring structures and regional lymph nodes (stage II and III) is the histological type a determining factor in survival.

### REFERENCES

Ashley, D. J. B. & Davies, H. D. (1967) Cancer of the Lung—Histology and Biological Behavior. *Cancer, N.Y.*, **20**, 165.

Bennet, D. E. & Sasser, W. F. (1969) Bronchiolar Carcinoma: a Valid Clinico-pathological Entity? A Study of 30 Cases. *Cancer, N.Y.*, **24**, 876.

Bergson, J. & Gage, R. P. (1950) Calculation of Survival Rates for Cancer. *Proc. Mayo Clin.*, **25**, 270.

Berrino, F., Musso, M. & Campobasso, O. (1971) Pathological Factors in Survival of Lung Tumours: Local Extent, Size and Nodal Involvement. *Br. J. Cancer*, **25**, 669.

Campobasso, O. (1968) The Characteristics of Peripheral Lung Tumours that Suggest Their Bronchiolo-alveolar Origin. *Br. J. Cancer*, **22**, 655.

Campobasso, O., Musso, M. & Berrino, F. (1970) Categorie istopatologiche di estensione locale dei tumori del polmone. *Tumori*, **56**, 223.

Denoix, P. (1969) The Determination and Expression of Cancer Survival Rates. In *TNM-General Rules*. Geneva: U.I.C.C.

Galfore' H., Payne, W. S., Woolner, L. B., Clagett, O. T. & Cage, R. P. (1964) Pathologic Classification and Surgical Treatment of Bronchogenic Carcinoma. *Surgery Gynec. Obstet.*, **119**, 51.

Hukill, P. B. & Stern, H. (1962) Adenocarcinoma of the Lung: Histological Factors Affecting Prognosis. *Cancer., N.Y.*, **15**, 504.

Jackman, R. J., Good, C. A., Clagett, O. T. & Woolner, L. B. (1969) Survival Rates in Peripheral Bronchogenic Carcinomas up to Four Centimeters in Diameter Presenting as Solitary Pulmonary Nodules. *J. thorac. cardiovasc. Surg.*, **57**, 1.

Jones, J. C., Kern, W. H., Chapman, N. D., Meyer, B. W. & Linde-Smith, G. G. (1967) Long-term Survival after Surgical Resection for Bronchogenic Carcinoma. *J. thorac. cardiovasc. Surg.*, **54**, 383.

Kern, W. H., Jones, J. C. & Chapman, N. D. (1968) Pathology of Bronchogenic Carcinoma in Long-term Survivors. *Cancer, N.Y.*, **21**, 772.

Kirsh, M. V., Prior, M., Gago, O., Moores, W. Y., Kahn, D. R., Pellegrini, R. V. & Sloan, H. (1972) The Effect of Histological Cell Type on the Prognosis of Patients with Bronchogenic Carcinoma. *Ann. thorac. Surg.*, **13**, 303.

Kreyberg, L., Liebow, A. A. & Uehlinger, E. A. (1967) *Histological Typing of Lung Tumours*. Geneva: W.H.O.

Lennox, S. C., Flavell, G., Pollock, D. J., Thompson, V. C. & Wilkins, J. L. (1968) Results of Resection for Oat-cell Carcinoma of the Lung. *Lancet*, ii, 925.

Mantel, N. (1966) Evaluation of Survival Data and Two Rank Order Statistics Arising in its Consideration. *Cancer chemother. Rep.*, **50**, 163.

Mollo, F., Canese, M. G. & Campobasso, O. (1973) Human Peripheral Lung Tumours: Light and Electron Microscopic Correlation. *Br. J. Cancer*, **27**, 173.

Mottura, G. & Campobasso, O. (1966) Sui criteri morfologici e istogenetici per la classificazione dei carcinomi broncopolmonari. *Minerva pneumol.*, **5**, 40.

Slack, N. H. (1970) Bronchogenic Carcinoma: Nitrogen Mustard as a Surgical Adjuvant and Factors Influencing Survival. University Surgical Adjuvant Lung Project. *Cancer, N.Y.*, **25**, 987.

Sobin, L. H. (1972) Multiplicity of Lung Tumours Classifications. *Recent Results Cancer Res.*, **39**, 29.

Weiss, W. (1971) Peripheral Measureable Bronchogenic Carcinoma. Growth Rate and Period of Risk after Therapy. *Am. Rev. resp. Dis.*, **103**, 198.

Weiss, W., Boucot, K. R. & Cooper, D. A. (1970) The Histopathology of Bronchogenic Carcinoma and its Relation to Growth Rate, Metastases and Prognosis. *Cancer, N.Y.* **26**, 965.